# Neurological soft signs in neurodegenerative dementias: Results of the DemeNSS study

**DOI:** 10.1002/pcn5.70143

**Published:** 2025-06-25

**Authors:** Federico Emanuele Pozzi, Anna Falco, Gaia Gotti, Giuseppe Fiamingo, Giulia Remoli, Ildebrando Appollonio, Carlo Ferrarese, Lucio Tremolizzo

**Affiliations:** ^1^ Neurology Department Fondazione IRCCS San Gerardo dei Tintori Monza Italy; ^2^ Milan Center for Neuroscience (NeuroMI) University of Milano‐Bicocca Milan Italy; ^3^ School of Medicine and Surgery University of Milano‐Bicocca Milan Italy

**Keywords:** Alzheimer' disease, clinical neurology, cognition, dementia, neurodegeneration

## Abstract

**Aim:**

Neurological soft signs (NSSs) encompass subtle neurological abnormalities, often indicative of impaired motor and sensory integration, observed in various neuropsychiatric conditions. NSSs have been recently investigated as potential diagnostic markers in neurodegenerative dementias. We aimed to confirm an NSS increase in subjects with cognitive decline and evaluate them in the differential diagnosis of neurodegenerative dementias.

**Methods:**

A sample of 93 subjects with dementia (34 with Alzheimer's disease [AD], 29 with frontotemporal dementia [FTD], 16 with Lewy body disease [LBD], and 14 with corticobasal syndrome [CBS]) and 93 healthy controls (HCs) were assessed using the 16‐item Heidelberg NSS Scale.

**Results:**

Subjects with neurodegenerative dementias exhibited significantly higher NSS scores than HCs (20.4 ± 7.9 vs. 5.7 ± 4.2, *p* < 0.01). Notably, those with CBS/LBD showed markedly elevated NSSs compared to those with AD and FTD (26.2 ± 6.7 vs. 18.4 ± 7.1 and 16.6 ± 6.5, respectively, *p* < 0.01). Diagnosis, Mini‐Mental State Examination (MMSE), Frontal Assessment Battery, and anticholinergic burden were significant predictors of NSS expression in subjects with dementia. In HCs, only age and MMSE were significant predictors. A reduced Neurological Soft Signs (rNSS) Scale, including only five items that can be administered in less than a minute, demonstrated diagnostic performances comparable to the full NSS Scale.

**Conclusion:**

NSSs are increased across neurodegenerative dementia subtypes, particularly in CBS and LBD. The Heidelberg NSS Scale, as well as its variant rNSS, may serve as quick and informative tools to be added to the visits in memory clinics.

## Graphical abstract

Neurological soft signs (NSSs), subtle motor and sensory abnormalities found in psychiatric disorders, are elevated in neurodegenerative dementias, particularly in corticobasal syndrome and Lewy body disease compared to Alzheimer's disease and frontotemporal dementia. A 5‐item NSS Scale performed comparably to the full Heidelberg NSS Scale, highlighting its potential as a rapid screening tool in memory clinics.

## INTRODUCTION

Neurological soft signs (NSSs) are subtle sensory–motor abnormalities, the biological significance of which is still a matter of debate.^1^ NSSs span different domains often overlooked during standard neurological examination, including motor coordination and sensory integration. Even when these aspects are investigated, they are usually not part of any of the many scales developed to quantify NSS expression.^2^ An elevated NSS expression has historically been linked to psychiatric disorders and has been long considered an endophenotype of psychosis. In 1975, Tucker and colleagues introduced the term NSSs after the identification of an increase in semiotic abnormalities in a sample of 109 subjects with a first psychotic episode, compared with healthy controls (HCs).^3^ Subsequent studies demonstrated an increased NSS expression even in healthy relatives of subjects with psychosis.^4^

Nevertheless, NSS increased burden has been shown in other psychiatric disturbances, including obsessive–compulsive disorder, borderline personality disorder, and attention deficit hyperactivity disorder.^5^, ^6^, ^7^ This has undermined the specificity of NSSs, supporting the possibility that they might be the expression of traits shared among different psychiatric conditions.

Moreover, these signs, long considered to be non‐localizing, might be indeed partly localizable.^1^ Studies on healthy subjects show that they may be related to a cerebellar contribution^8^ and variations in microstructure within sensory–motor cortical and subcortical white matter.^9^ We previously reported a significant increase in NSS expression in migraine subjects compared to non‐cephalalgic subjects. In migraine, NSS expression was higher in subjects with deep white matter hyperintensities.^10^, ^11^

Several authors have investigated NSS expression in relation to age, mild cognitive impairment (MCI) and Alzheimer's disease (AD), as well as genetic risk factors for dementia, such as APOE ε4.^12^, ^13^, ^14^, ^15^, ^16^, ^17^ Behavioral and psychological symptoms of dementia occur in up to 90% of patients with dementia, correlating with the severity of cognitive and functional impairment.^18^, ^19^ This pervasive presence of psychiatric abnormalities might be the rationale for NSS expression in dementias, with an increase throughout the disease progression. However, an association with antipsychotic therapy has not been demonstrated yet.^16^, ^17^

In a recent communication at the 2021 World Congress of Neurology, our group confirmed an increase of NSSs in AD subjects, and reported preliminary evidence for such an increase also in subjects with other neurodegenerative dementias. Albeit increased across all diagnoses, NSS expression was milder in frontotemporal dementia (FTD) and maximal in dementia with Lewy bodies (LBD) and corticobasal syndrome (CBS). AD subjects exhibited an intermediate NSS profile.^20^ In this pilot work we used the modified Heidelberg NSS Scale, comprising only a few NSS items, and thus possibly less discriminant. Moreover, we could not individuate specific disease‐associated NSS patterns. Finally, the sample comprised mostly subjects in moderate phases of dementia. Based on these data, our aim was to study NSS expression in neurodegenerative dementias with the full Heidelberg NSS Scale.

## METHODS

### Pilot work

The sample was comprised of 29 HCs (with the same criteria listed below) and 85 subjects with neurodegenerative dementias. Of these subjects, 62 were affected by AD, 10 by FTD and 13 by either CBS or LBD. In this cohort, we only tested the modified Heidelberg NSS Scale (which we call here rNSS‐2, range: 0–18), used in previous work on NSSs and dementia.^16^, ^17^ The rNSS‐2 included the following signs: finger‐to‐nose, diadochokinesia, prono‐supination, thumb‐fingers opposition (for each hand), and mirror movements. The results of the pilot work are available in the Tables S1–S5 and Figures S1–S7.

### Main study: Subject selection and procedures

Following ethical committee approval (Monza e Brianza, Italy; protocol DemeNSS), we planned to enroll a novel cohort of 90 consecutive subjects affected by AD (*n* = 30), FTD (*n* = 30), and CBS or LBD (*n* = 30). Subjects were selected among outpatients visiting our Center for Dementia and Cognitive Decline at Fondazione IRCCS San Gerardo dei Tintori, Monza.

Inclusion criteria were the following:

a) diagnosis of AD according to the National Institute of Aging and Alzheimer's Association 2011 criteria^21^; FTD according to Rascovsky (behavioral variant) or Gorno‐Tempini (primary progressive aphasia, semantic and non‐fluent variants) criteria^22^, ^23^; CBS according to Armstrong criteria^24^; or probable LBD according to McKeith 2017 criteria^25^;
b) Mini‐Mental State Examination (MMSE) > 10;
c) age > 40 years;
d) signed informed consent.

Exclusion criteria were:

a) mixed forms of dementia with a prevalent vascular component, or vascular dementia;
b) relevant neurologic or psychiatric comorbidities;
c) substance or alcohol abuse.

An MMSE cut‐off of 10 was selected based on a ceiling effect of NSSs around this value on rNSS‐2 in the pilot study (see Material S1).^20^ Moreover, we argued that it was probably futile to evaluate diagnostic properties of NSSs in subjects with severe dementias, as the specific etiological diagnosis is less relevant in this phase.

All subjects underwent NSS evaluation, carried out by two trained neurologists, in a calm environment, without interruptions or other observers. We used the 16‐item Heidelberg NSS Scale.^26^ Each item is rated 0–3 (total range: 0–48). We chose this scale since, unlike other NSS batteries, it excludes primitive reflexes, more properly considered as markers of cognitive and upper motor neuron dysfunctions.^27^ We did not differentiate between left and right, but rather used the greatest value between the two sides to avoid giving more weight to items assessed bilaterally. The Italian version is available in the Material S1.

From the full Heidelberg NSS Scale, we extracted two reduced NSS scales: the rNSS Scale, obtained by selecting the five items significantly worse in subjects with CBS or LBD compared to subjects with AD or FTD, and the aforementioned rNSS‐2.^20^

Subjects also underwent MMSE and the frontal assessment battery (FAB).^28^ Caregivers were asked to complete activities of daily living (ADL) and instrumental activities of daily living (IADL) questionnaires, as well as the Neuropsychiatric Inventory (NPI),^29^ to evaluate whether NSS burden correlated with psychiatric burden or whether it represented an independent phenotype associated with the neurodegenerative disorder.

We also gathered data on familial history of dementia, education, drugs (in order to calculate anti‐cholinergic burden [ACB) and equivalents of olanzapine, fluoxetine and diazepam^30^, ^31^, ^32^, ^33^) and comorbidities.

HCs were recruited among relatives of outpatients seen for reasons other than dementias in our neurology clinic. They were matched for sex, age (±4 years) and education (±4 years) with enrolled patients. Exclusion criteria for HCs were any history of neurological or psychiatric disorder and treatment with psychoactive drugs. HCs were enrolled after signing informed consent.

### Sample size calculation and statistical analysis

Sample size was calculated considering a clinically relevant difference of at least 4.5 points between patients and HCs, with an effect size of 0.40 based on preliminary observations. With an independent samples *t*‐test, considering alpha 0.05 and beta 0.20, sample size was calculated at 180 subjects (90 patients and 90 HCs). Sample size calculation was performed with G*Power 3.126.^34^

Statistical analysis was performed with R. Data are reported as mean ± standard deviation. Differences between patients and HC were evaluated with a *t*‐test. Multivariable linear models were run to test the effect of explanatory variables on NSS expression. Pearson product–moment correlation was used to test correlation between variables.

## RESULTS

### Baseline characteristics

The final samples comprised 93 patients and 93 HCs. Among subjects with dementia, we enrolled 34 with AD, 29 with FTD, 16 with LBD and 14 with CBS (the latter two grouped together under the “subcortical dementias” umbrella due to their parkinsonian features).

Baseline differences between HCs and the whole sample are shown in Table 1. As expected, HCs had significantly higher MMSE and FAB scores compared to patients (29.0 ± 1.4 vs 20.4 ± 5.3 and 16.3 ± 1.9 vs 9.2 ± 3.3, respectively, *p* < 0.01).

**Table 1:** Baseline differences between patients and HCs.

Baseline variable	HCs (*n* = 93)	Patients (*n* = 93)	*p*
Age	75.0 ± 7.5	74.8 ± 7.1	ns
Female sex	60 (65%)	60 (65%)	ns
Education	8.3 ± 3.5	7.8 ± 3.5	ns
MMSE	29.0 ± 1.4	20.4 ± 5.3	<0.01
FAB	16.3 ± 1.9	9.2 ± 3.3	<0.01
NSS	5.7 ± 4.2	20.4 ± 7.9	<0.01
rNSS	1.0 ± 1.1	5.0 ± 3.4	<0.01
rNSS‐2	0.8 ± 1.1	5.2 ± 4.2	<0.01

Abbreviations: FAB, Frontal Assessment Battery; HCs, healthy controls; ns, not significant; NSS, Neurological Soft Signs Scale; rNSS, reduced Neurological Soft Signs Scale; rNSS‐2, reduced Neurological Soft Signs Scale (pilot study version).

Baseline differences among the three diagnostic groups are summarized in Table 2. Subjects with FTD were younger than those with other conditions (FTD: 71.0 ± 6.5 years, AD: 77.4 ± 6.2 years, CBS or LBD: 75.5 ± 7.4 years; *p* < 0.01). Significant differences were present among dementia groups in terms of MMSE, with FTD exhibiting higher values compared to AD or CBS/LBD. No differences were noted on the FAB.

**Table 2:** Baseline differences among diagnostic groups.

Baseline variables	FTD (*n* = 29)	AD (*n* = 34)	CBS or LBD (*n* = 30)	*p*
Age	71.0 ± 6.5	77.4 ± 6.2	75.5 ± 7.4	<0.01
Female sex	16 (55%)	26 (76%)	18 (60%)	ns
Education (years)	8.8 ± 4.0	7.3 ± 3.3	7.4 ± 3.2	ns
Diazepam equivalents (mg)	0.7 ± 2.3	0.2 ± 1.1	0.0 ± 0.0	ns
Olanzapine equivalents (mg)	0.7 ± 2.6	0.1 ± 0.4	0.6 ± 2.6	ns
Fluoxetine equivalents (mg)	4.1 ± 9.1	6.2 ± 10.0	5.3 ± 9.2	ns
MMSE	22.5 ± 5.4	19.6 ± 5.4	19.2 ± 4.7	<0.05
FAB	9.7 ± 3.7	9.1 ± 3.2	8.9 ± 3.1	ns
NSS	16.6 ± 6.5	18.4 ± 7.1	26.2 ± 6.7	<0.01
rNSS	3.7 ± 2.5	3.4 ± 2.6	8.0 ± 2.9	<0.01
rNSS‐2	3.9 ± 3.7	4.0 ± 3.4	7.8 ± 4.3	<0.01
NPI	31.3 ± 23.3	19.5 ± 19.5	35.2 ± 30.3	<0.05
ADL	5.0 ± 1.4	5.0 ± 1.6	4.7 ± 1.5	ns
IADL	5.2 ± 2.8	4.9 ± 2.9	3.7 ± 2.4	ns

Abbreviations: AD, Alzheimer's disease; ADL, activities of daily living; CBS, corticobasal syndrome; FAB, Frontal Assessment Battery; FTD, frontotemporal dementia; IADL, instrumental activities of daily living; LBD, Lewy body disease; MMSE, Mini‐Mental State Examination; NPI, Neuropsychiatric Inventory; ns, not significant; NSS, Neurological Soft Signs Scale; rNSS, reduced Neurological Soft Signs Scale; rNSS‐2, reduced Neurological Soft Signs Scale (pilot study version).

There were no statistically significant differences among diagnostic groups in terms of comorbidities, psychoactive drugs equivalents, ACB, or disease duration (data not shown). Diabetes was present in 12% (*n* = 11) of our sample. Mean ACB was 1.0 ± 1.4. Regarding psychoactive drugs, 14% (*n* = 13) of patients were on benzodiazepines (diazepam equivalents 0.3 ± 1 mg), 9% (*n* = 8) were on antipsychotics (olanzapine equivalents 0.4 ± 0.2 mg), and 25% (*n* = 23) were on antidepressants (fluoxetine equivalents 5 ± 9 mg). Only 8% (*n* = 7) and 4% (*n* = 4) of patients were on acetylcholinesterase inhibitors or memantine.

### NSS expression

On the full Heidelberg NSS Scale, the mean NSS score was significantly increased in patients compared to HCs (20.4 ± 7.9 vs 5.7 ± 4.2, *p* < 0.01). The same was true also for the mean scores on the two reduced NSS scales (see Table 1). All NSS items were significantly higher in patients compared to HCs (data not shown).

**Figure 1:**
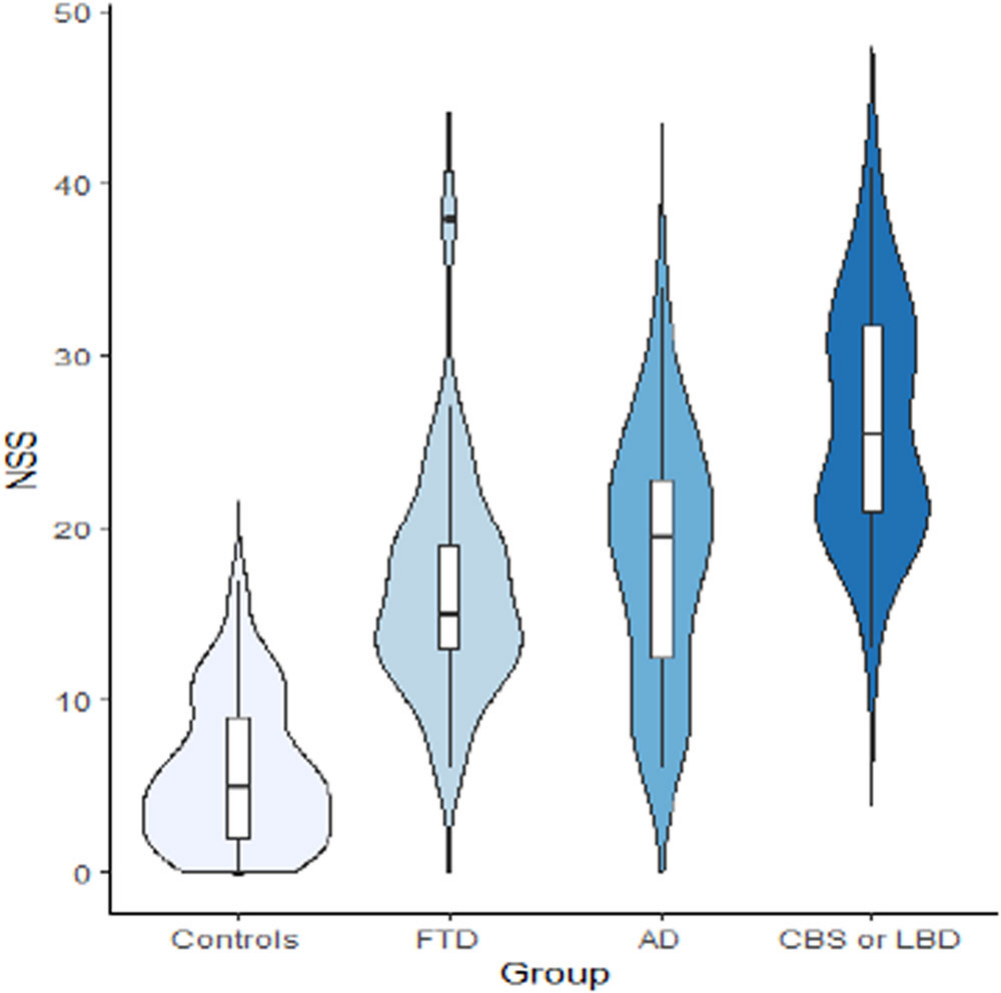
NSS expression across diagnostic categories with the full Heidelberg Neurological Soft Sign (NSS) Scale. AD, Alzheimer's disease; CBS, corticobasal syndrome; FTD, frontotemporal dementia; LBD, Lewy body disease.

In addition, all three mean NSS scores were higher in patients with CBS or LBD compared to those with AD or FTD (FTD: 16.6 ± 6.5, AD: 18.4 ± 7.1, CBS or LBD: 26.2 ± 6.7; *p* < 0.01, see Table 2 and Figure 1). Importantly, even the latter two groups had significantly higher NSS scores compared to HCs. The items that were significantly worse in subjects with CBS or LBD compared to those with AD or FTD included evaluation of gait, walking on a straight line (one foot in front of the other), finger‐to‐nose, diadochokinesia, and prono‐supination (see Table 3). These five items were grouped into the rNSS Scale (total range: 0–15), which could be administered in less than 1 min.

**Table 3:** Baseline differences in single NSS items among diagnostic groups.

NSS items	FTD (*n* = 29)	AD (*n* = 34)	CBS or LBD (*n* = 30)	*p*
**Gait**	0.3 ± 0.5	0.3 ± 0.6	**1.2** ± **0.8**	<0.01
**Walking on a straight line**	1.2 ± 1.0	1.3 ± 1.1	**2.2** ± **0.7**	<0.01
Right‐Left touches	1.6 ± 1.2	2.0 ± 1.3	1.9 ± 1.3	ns
Straight arms	0.2 ± 0.4	0.0 ± 0.0	0.2 ± 0.4	<0.05
**Finger to nose**	0.7 ± 0.8	0.4 ± 0.7	**1.4** ± **1.2**	<0.01
Ozeretzki	2.2 ± 0.7	2.6 ± 0.6	2.8 ± 0.5	<0.01
**Diadochokinesia**	0.9 ± 1.1	0.7 ± 1.0	**1.7** ± **1.1**	<0.01
**Prono‐supination**	0.6 ± 0.9	0.7 ± 1.1	**1.3** ± **1.2**	<0.05
Thumb‐finger opposition R	0.5 ± 0.7	0.8 ± 1.1	1.3 ± 1.0	<0.01
Thumb‐finger opposition L	0.6 ± 0.8	0.9 ± 1.0	1.3 ± 1.0	<0.05
Mirror movements	0.5 ± 0.8	0.5 ± 0.7	0.8 ± 0.8	ns
Two points discrimination	1.6 ± 1.2	2.1 ± 1.2	2.4 ± 1.0	<0.05
Graphesthesia	1.0 ± 1.1	1.5 ± 1.1	1.8 ± 0.9	<0.05
Face–hand touches	1.0 ± 0.9	1.0 ± 1.0	1.6 ± 1.0	<0.05
Stereognosis	1.3 ± 1.1	1.3 ± 1.1	1.8 ± 1.0	ns
Luria sequence	1.0 ± 1.1	1.6 ± 1.0	1.8 ± 1.0	<0.01
Tongue‐twisters	1.8 ± 1.0	1.6 ± 1.1	1.9 ± 1.1	ns

*Note*: Values in bold represent significantly higher values in CBS/LBD patients compared with both AD and FTD patients.

Abbreviations: AD, Alzheimer's disease; CBS, corticobasal syndrome; FTD, frontotemporal dementia; LBD, Lewy body disease; ns, not significant; NSS, Neurological Soft Signs Scale.

**Figure 2:**
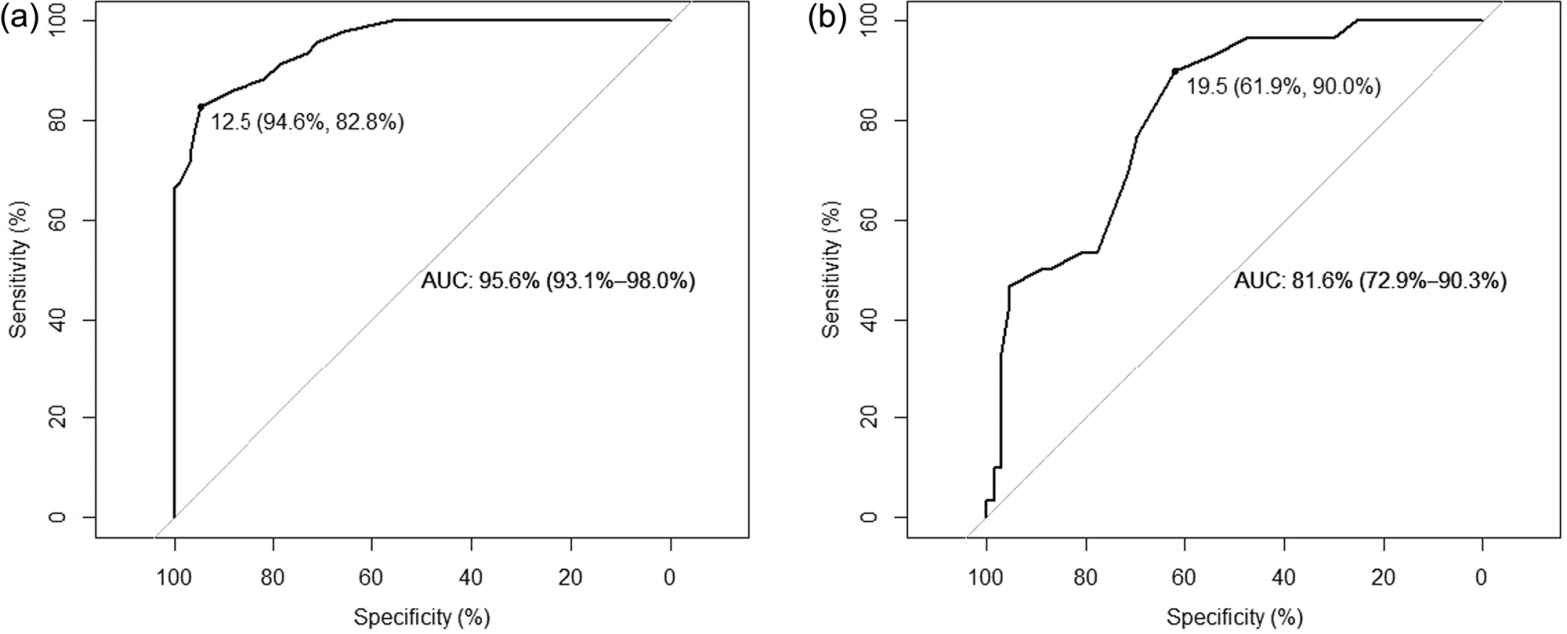
Receiver operating characteristic (ROC) curves for neurological soft signs (NSSs) performances in differentiating (a) patients from healthy controls and (b) patients with corticobasal syndrome (CBS)/Lewy body disease (LBD) from those with Alzheimer's disease (AD)/frontotemporal dementia (FTD) on the full Heidelberg NSS Scale.

We performed a receiver operating characteristic (ROC) analysis of the NSS scales. For the diagnosis of neurodegenerative dementia, we obtained an excellent area under the curve (AUC) of 95.6 (95% confidence interval [CI]: 93.1–98.0) with the full NSS Scale. A cut‐off of 12.5 points allowed discrimination between patients with neurodegenerative dementias and HCs with a 94.6% sensitivity and an 82.8% specificity (Figure 2a). For the differential diagnosis between CBS/LBD and other dementias, the AUC was 81.6 (95% CI: 72.9–90.3). A cut‐off of 19.5 yielded a 61.9% sensitivity, and an excellent 90.0% specificity (Figure 2b). The discriminative power between CBS and LBD was poor, possibly due to low sample size.

**Figure 3:**
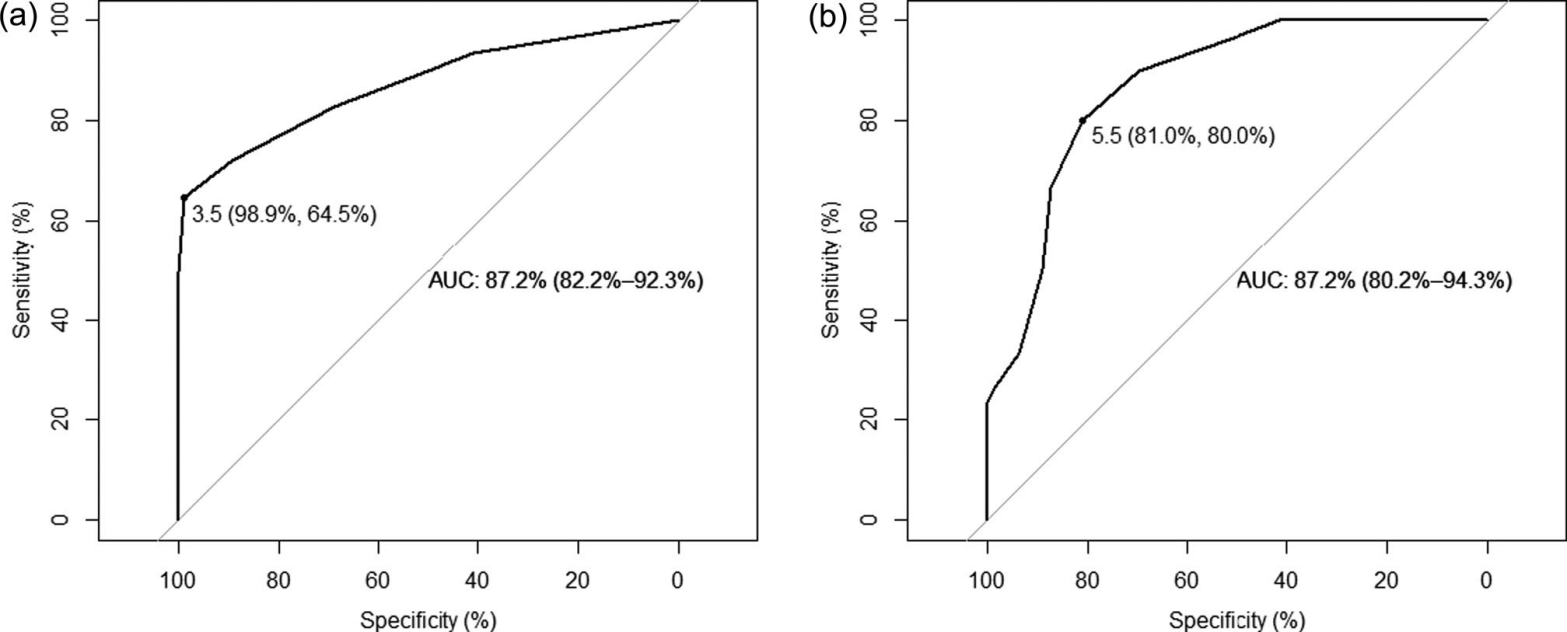
Receiver operating characteristic (ROC) curves for neurological soft signs (NSSs) performances in differentiating (a) patients from healthy controls and (b) patients with corticobasal syndrome (CBS)/Lewy body disease (LBD) from those with Alzheimer's disease (AD)/frontotemporal dementia (FTD) on the reduced Neurological Soft Signs (rNSS) Scale.

With the rNSS Scale, a cut‐off of 3.5 discriminated between neurodegenerative dementias and HC with an excellent sensitivity (98.9%), although only poor specificity (64.5%); the AUC was 87.2 (95% CI: 82.2–92.3, Figure 3a). A cut‐off of 5.5 yielded good sensitivity and specificity for the diagnosis of CBS or LBD versus other dementias (81.0% and 80.0% respectively, AUC 87.2, 95% CI: 80.2–94.3, Figure 3b). The rNSS‐2 performances were a bit worse (data not shown).

We ran three groups of multivariable linear models to test predictors of NSS expression with the three different scales. The first group of models was applied to the whole sample and included group (patients vs HC), age, education, sex, MMSE, and FAB scores as predictors. All models were statistically significant, with good adjusted *R* ^2^, explaining at least 44% of the variance in NSSs with all three scales. MMSE was the only consistent predictor across all three scales, with an inverse relationship with NSS expression. Being diagnosed with a neurodegenerative dementia predicted higher NSS scores on the full scale and on rNSS (but not on rNSS‐2). Age was a significant negative predictor only for rNSS‐2, in that older age predicted lower NSS expression. Higher FAB scores predicted a lower NSS expression only with the full scale. Model summaries are reported in Table 4.

**Table 4:** Linear models for NSS expression (whole sample).

	NSS		rNSS		rNSS‐2	
*Predictors*	*Estimates*	*p*	*Estimates*	*p*	*Estimates*	*p*
(Intercept)	33.13 (21.87–44.39)	**<0.001**	8.13 (2.75–13.51)	**0.003**	16.00 (9.64–22.36)	**<0.001**
Group [patients]	4.26 (1.71–6.81)	**0.001**	1.83 (0.61–3.05)	**0.004**	1.19 (−0.25–2.63)	0.106
Age	0.04 (−0.07–0.15)	0.427	−0.00 (−0.06–0.05)	0.917	−0.07 (−0.13–−0.01)	**0.022**
Education	−0.07 (−0.30–0.17)	0.574	−0.01 (−0.12–0.11)	0.929	0.02 (−0.11–0.15)	0.743
Sex [F]	0.13 (−1.37–1.64)	0.862	0.09 (−0.63–0.81)	0.808	0.19 (−0.66–1.04)	0.656
MMSE	−0.70 (−0.92–−0.48)	**<0.001**	−0.22 (−0.33–−0.12)	**<0.001**	−0.30 (−0.42–−0.18)	**<0.001**
FAB	−0.61 (−0.93–−0.29)	**<0.001**	−0.03 (−0.18–0.13)	0.736	−0.09 (−0.27–0.09)	0.340
*R* ^2^	0.747		0.467		0.464	
Adjusted *R* ^2^	0.738		0.449		0.446	
*p* (model)	<0.001		<0.001		<0.001	

Abbreviations: FAB, Frontal Assessment Battery; MMSE, Mini‐Mental State Examination; NSS, Neurological Soft Signs Scale; rNSS, reduced Neurological Soft Signs Scale; rNSS‐2, reduced Neurological Soft Signs Scale (pilot study version).

The second group of models was only applied to subjects with neurodegenerative dementias, testing diagnosis (coded as such: FTD = 1, AD = 2, CBS or LBD = 3, based on the differences observed in NSS expression), age, ACB, psychoactive drugs equivalents, MMSE, FAB, and NPI. All models were statistically significant, with good adjusted *R* ^2^, albeit lower than the first group of models. Diagnosis was a significant predictor of NSS expression with all scales. MMSE was again a consistent negative predictor of NSS expression, while higher FAB and fluoxetine equivalents predicted a lower NSS expression only with the full scale. Age represented a significant negative predictor only for rNSS‐2. Interestingly, ACB was a consistent predictor across all scales, with higher values corresponding to higher NSS expression. Model summaries are reported in Table 5.

**Table 5:** Linear models for NSS expression (only patients with neurodegenerative dementias).

	NSS		rNSS		rNSS‐2	
*Predictors*	*Estimates*	*p*	*Estimates*	*p*	*Estimates*	*p*
(Intercept)	34.01 (19.04–48.97)	**<0.001**	7.72 (0.35–15.09)	**0.040**	14.24 (4.40–24.08)	**0.005**
Diagnosis	4.25 (2.62–5.87)	**<0.001**	2.13 (1.33–2.93)	**<0.001**	1.97 (0.90–3.04)	**<0.001**
Age	−0.09 (−0.27–0.08)	0.281	−0.06 (−0.14–0.03)	0.195	−0.12 (−0.23–−0.00)	**0.044**
ACB	2.08 (0.59–3.57)	**0.007**	0.74 (0.00–1.47)	**0.050**	1.41 (0.43–2.39)	**0.006**
Olanzapine eq.	−0.26 (−1.00–0.49)	0.496	0.10 (−0.26–0.47)	0.573	−0.20 (−0.70–0.29)	0.411
Diazepam eq.	0.12 (−0.74–0.99)	0.775	−0.03 (−0.45–0.40)	0.901	−0.33 (−0.90–0.24)	0.251
Fluoxetine eq.	−0.16 (−0.31–−0.00)	**0.045**	−0.05 (−0.13–0.02)	0.171	−0.01 (−0.12–0.09)	0.770
MMSE	−0.51 (−0.78–−0.23)	**<0.001**	−0.16 (−0.29–−0.02)	**0.025**	−0.21 (−0.39–−0.03)	**0.023**
FAB	−0.74 (−1.18–−0.31)	**0.001**	−0.07 (−0.29–0.14)	0.503	−0.14 (−0.43–0.14)	0.322
NPI	0.03 (−0.02–0.08)	0.207	0.02 (−0.01–0.04)	0.132	0.01 (−0.02–0.05)	0.389
*R* ^2^	0.645		0.497		0.439	
Adjusted *R* ^2^	0.600		0.433		0.367	
*p* (model)	<0.001		<0.001		<0.001	

Abbreviations: ACB, anticholinergic burden; FAB, Frontal Assessment Battery; MMSE, Mini‐Mental State Examination; NPI, Neuropsychiatric Inventory; NSS, Neurological Soft Signs scale; rNSS, reduced Neurological Soft Signs scale; rNSS‐2, reduced Neurological Soft Signs scale (pilot study version).

Finally, we ran linear models to test predictors of NSS expression in HCs. Age was a significant positive predictor of NSSs on the full NSS Scale and rNSS, but not on rNSS‐2. MMSE was a significant negative predictor of NSS expression, while FAB was a significant negative predictor only with the full NSS Scale (Table 6). NSS expression in HCs did not differ according to familial history of dementia (*p* = 0.99).

**Table 6:** Linear models for NSS expression in healthy controls.

	NSS		rNSS		rNSS‐2	
*Predictors*	*Estimates*	*p*	*Estimates*	*p*	*Estimates*	*p*
(Intercept)	33.43 (15.45–51.41)	**<0.001**	5.16 (0.20–10.12)	**0.041**	12.86 (7.48–18.23)	**<0.001**
Age	0.13 (0.03–0.23)	**0.013**	0.04 (0.01–0.07)	**0.004**	−0.02 (−0.05–0.02)	0.316
Sex [1]	1.15 (−0.32–2.62)	0.124	0.32 (−0.08–0.73)	0.118	0.32 (−0.12–0.76)	0.155
Education	0.05 (−0.18–0.28)	0.661	0.01 (−0.05–0.08)	0.693	0.06 (−0.01–0.13)	0.104
MMSE	−1.06 (−1.66–−0.45)	**0.001**	−0.27 (−0.44–−0.10)	**0.002**	−0.35 (−0.53–−0.16)	**<0.001**
FAB	−0.50 (−0.94– −0.06)	**0.028**	0.01 (−0.11–0.14)	0.816	−0.10 (−0.23–0.04)	0.153
*R* ^2^	0.402		0.275		0.265	
Adjusted *R* ^2^	0.367		0.234		0.223	
*p* (model)	<0.001		<0.001		<0.001	

Abbreviations: FAB, Frontal Assessment Battery; MMSE, Mini‐Mental State Examination; NSS, Neurological Soft Signs scale; rNSS, reduced Neurological Soft Signs scale; rNSS‐2, reduced Neurological Soft Signs scale (pilot study version).

MMSE and FAB showed a good negative correlation with NSS expression on the full scale (*r* = −0.81 and *r* = −0.80, respectively, *p* < 0.001). Correlations of MMSE and FAB with NSS on the other scales were still significant, albeit only moderately (data not shown). Education showed a small negative correlation with NSS expression only with the full scale (*r* = −0.21, *p* < 0.01). All NSS scales exhibited a high correlation between each other (NSS–rNSS *r* = 0.86, NSS–rNSS‐2 *r* = 0.85, rNSS–rNSS‐2 *r* = 0.86, *p* < 0.001).

## DISCUSSION

In the present study, we confirmed an increased NSS expression in patients affected by neurodegenerative dementias compared to HCs with all tested with the NSS Scale. NSS expression seems higher in patients with CBS or LBD compared to those with AD or FTD; this was replicated in two distinct cohorts. We developed a new reduced NSS Scale (rNSS), with diagnostic performances comparable to the full NSS Scale and the previously used reduced scale (rNSS‐2^20^). We also provided optimal cut‐offs with good sensitivity for the diagnosis of neurodegenerative dementias and good specificity for the differential diagnosis of CBS or LBD.

Motor dysfunction is recognizable across all forms of dementia compared to HCs.^35^, ^36^ Most of the items included in the Heidelberg NSS Scale (and especially in the rNSS) involve motor function, and greatly depend on cerebellar function or subcortical sensorimotor integration,^37^ which seems consistent with brain correlates of NSSs. It is hard to find specific brain correlates for signs that are part of a non‐localizable semiology. Nevertheless, previous research has tried to investigate whether structural or functional brain abnormalities may be related to NSSs in various clinical populations and healthy adults, finding widespread alterations in cerebello‐thalamo‐basal‐frontal areas.^38^, ^39^, ^40^, ^41^, ^42^ Studies on individuals with “ultra‐high” risk for psychosis found an association between NSS expression and decreased grey matter volumes in the frontal cortex, insula, caudate, and cerebellum.^43^, ^44^ Another study on healthy subjects revealed an association between NSSs and structural network abnormalities in cerebellar, subcortical and cortical sensorimotor areas.^45^ NSSs are also present in organic brain disease, such as HIV‐associated neurocognitive disorder, in which they are associated with grey matter reductions in the insula and cerebellum. Interestingly, NSSs are increased in HIV‐associated neurocognitive disorder compared to HIV patients without cognitive impairment, and are associated with abnormalities in the same areas.^46^, ^47^, ^48^

The fact that NSSs are increased across such a heterogeneous set of clinical and preclinical neuropsychiatric conditions,^49^, ^50^ and their consistent association with the same network, might be a good argument against a separation of “psychiatric” and “organic” diseases. As such, NSSs might represent a non‐specific signature of neuropsychiatric disorders in general, related to a dysfunction of cerebellar‐thalamic‐frontal circuits. These non‐localizable abnormalities, being indeed related to widespread “organic” alterations, albeit subtle, may make a good case for the idea that psychiatry is indeed neurology, and vice versa.^51^, ^52^, ^53^

The reason why CBS and LBD exhibit higher NSSs may be found in the greater involvement of sensorimotor areas and cerebellum compared to AD and FTD, as well as the differential involvement of cerebellum and subcortical structures.^54^, ^55^, ^56^ Indeed, the cerebellum seems affected by brain pathology in α‐synucleinopathies,^57^, ^58^ although cerebellar involvement may be demonstrated also in AD in the absence of overt neuropathology,^59^, ^60^ and a cerebellar and subcortical involvement, albeit less crucial and more heterogeneous across different disease genotypes, is also emerging in FTD.^61^, ^62^, ^63^, ^64^, ^65^, ^66^ Moreover, subjects with α‐synucleinopathies exhibit imbalances in connectivity among subcortical networks, cerebellum and frontoparietal networks,^67^ greatly overlapping with NSS brain correlates. Finally, the relative presence of extrapyramidal signs in all three scales (including at least five items that are also evaluated in the Unified Parkinson's Disease Rating Scale‐III^68^) might explain why subjects with expected parkinsonian features exhibit higher NSSs with the Heidelberg NSS Scale. Although less affected, AD and FTD are also known to exhibit motor abnormalities, such as gait disturbances and abnormal finger‐to‐nose test,^69^, ^70^ which may explain why all NSS scales separate these subjects from HCs. The rNSS, including proportionally more extrapyramidal signs compared to the full scale and rNSS‐2, may be even more specific for parkinsonian features and CBS/LBD diagnosis.

We found that treatment with psychoactive drugs did not influence NSS expression in patients with neurodegenerative diseases, perhaps with the exception of antidepressants (which were negative predictors of NSS only on the full scale). In a recent study, chlorpromazine equivalents did not influence NSS expression in patients with schizophrenia and predominantly negative symptoms.^71^ The same lack of influence of antipsychotics on NSSs was shown in adolescents at ultra‐high risk for psychosis.^72^ Neither antidepressants nor electroconvulsive therapy influenced NSS expression in major depressive disorder in another study.^73^

Interestingly, ACB was a significant and consistent predictor of NSS expression. This association was not investigated in previous research. It is well known that ACB results in worse neuropsychological performances in diverse clinical populations,^74^, ^75^, ^76^, ^77^, ^78^, ^79^ and the cholinergic system is pivotal in several neurodegenerative diseases.^80^ It seems that ACB does not influence progression of cognitive decline,^81^, ^82^ possibly acting as a reversible detrimental factor. On the other hand, ACB also affects motor function, being related to impaired gait in older adults.^83^, ^84^ Therefore, ACB may influence NSS expression through a double effect on motor function and cognition.

Age was a significant predictor of NSS expression with the full NSS Scale and with rNSS only in HCs, while in patients this was not significant. On the other hand, it was a significant negative predictor in patients and in the whole sample, but not in HCs, on rNSS‐2 (meaning that with increasing age, NSS expression on rNSS‐2 decreases). While we do not have a credible explanation for this association, the effect of age on NSS expression with the other two scales seems consistent with other studies. In particular, a recent paper investigated NSS with the full Heidelberg NSS Scale in healthy subjects, finding an increase in their expression with age.^85^ The results are not completely comparable to ours, as the authors scored each side on bilateral items in the NSS Scale independently (total NSS range: 0–81). Nevertheless, they reported a 2.4‐point increase in NSS every 10 years (1.3 in our case, total NSS range: 0–48), slightly greater in men than women. The most significant increase was observed between the sixth and seventh decade, and due to gait, tandem gait, Ozeretzki's test, finger‐thumb opposition, 2‐point discrimination and Luria's test, reflecting a worsening in motor tasks and sensory integration with age.^85^ Three of these signs were also present in rNSS‐2 (gait, tandem gait, finger‐thumb opposition, accounting for up to two‐thirds of the total points), while in rNSS only two of them are included (gait, tandem gait, with a total of six potential points out of 15). The authors included 60 subjects (20 per decade), while our sample of people aged 60 years and over were much more represented. In particular, we had only three subjects aged less than 60 years, 19 aged 60–69, 38 aged 70–79 and 33 aged 80+. In the study by Bachmann et al., a pairwise comparison of subjects aged 60–69 and 70+ did not reveal significant differences.^85^ Therefore, due to the different sample characteristics, our results possibly extend previous studies, showing that NSSs keep increasing in healthy subjects through the seventh to ninth decades, well beyond the period of most pronounced changes. Therefore, NSSs should probably not be regarded as stable in the elderly, as previously reported.^86^, ^87^

Contrary to some previous studies, including our own work in migraine patients, education did not predict NSS scores on any scale in our sample.^11^, ^17^ A few factors may explain this discrepancy. First, our multivariable linear models included a greater number of predictors compared to other studies, which may have attenuated the association between education and NSSs. For example, in the study by Urbanowitsch et al., the correlation between education and NSSs was statistically significant but modest (*r* = −0.25).^17^ Second, our sample had a generally lower level of education than those in prior studies (7–8 vs 12–14 years), which may suggest the existence of a threshold effect below which the association might not emerge. However, the literature on this point remains inconsistent, with some studies supporting a relationship between education and NSSs and others reporting no such association.^46^, ^47^, ^71^

Our study has a few limitations. A first limitation is the lack of standardization of NSS assessment. Indeed, several instruments are available to evaluate NSS, with a low mean overlap in terms of items (0.27 according to a recent studies).^88^ The 16‐item Heidelberg NSS Scale is widely used in studies with neuroimaging, accounting for 41% of publications, while it is rarely used in neuropsychological studies. Moreover, the Heidelberg NSS Scale includes fewer items compared to other scales, making it quicker to administer. It also shows optimal inter‐rater and test–retest reliability.^88^ Other scales range from 18 to 43 items,^89^, ^90^, ^91^, ^92^, ^93^ and exhibit worse reliability.^88^ While the Heidelberg NSS Scale seemed a reasonable choice to evaluate NSSs in patients with dementia, caution must be applied when generalizing results, as other scales may give different results.

Another limitation regards the way we treated diagnostic categories. We grouped together different forms of FTD, although they are notoriously heterogeneous both in terms of clinical presentation and brain involvement. This was primarily due to the relative rarity of FTD patients in our area, which hindered the recruitment of a large enough sample. Similarly, we grouped together CBS and LBD patients, which was justified by the presence of parkinsonism in both conditions, although they do not share other neuropathological or clinical affinities. On the contrary, the separation of CBS and LBD from AD may not be clear‐cut, as it is well known that half of cases of CBS are indeed sustained by AD pathology,^94^ and AD co‐pathology may be found in the majority of patients with LBD.^95^ Moreover, in our sample, biomarkers were not necessarily used for diagnosis, depending on criteria requirements. Nevertheless, the consistency of the findings across different cohorts (including also our preliminary work) may make these limitations less concerning.

While we provided NSS cut‐offs with good specificity and sensitivity for dementia diagnosis, our sample comprised subjects in moderate phases of dementia, in which the diagnosis is somewhat obvious. In this population, NSS clinical usefulness mostly lies in aiding in differential diagnosis between dementia forms. However, to be really useful in modern memory clinics, NSSs should differentiate between subjects at risk of or in the early phases of cognitive decline and HCs. It is well known that people at familial risk of schizophrenia exhibit NSSs that are intermediate between patients with schizophrenia and HCs.^4^ We did not find any difference in NSS expression according to familial history of dementia in HCs. Nevertheless, not many studies have investigated NSS expression in preclinical or prodromal phases of cognitive impairment, such as subjective cognitive decline or MCI.^96^, ^97^ One study reported that subjects with amnestic MCI exhibited higher NSSs compared to HCs, but the authors used a different NSS scale (the Cambridge Neurological Inventory),^14^ in contrast with another study that did not show differences between MCI and HCs with the rNSS‐2.^17^ A study by Lautenschlager et al. did not find differences in terms of NSSs between older adults with or without subjective cognitive complaints. Their results might not be directly comparable to other studies, as they built their own NSS scale. Interestingly, they showed higher NSS expression in APOE ε4 carriers, irrespective of cognitive complaints.^15^ Given the scarcity and heterogeneity of data, NSSs in patients with MCI and subjective cognitive decline represent an interesting future line of research.

Finally, the NSS scales correlated well with other cognitive measures, such as MMSE and FAB. This confirms previous research showing good associations between the NSS scales and cognition in healthy elderly.^13^ Therefore, considering all these data, the NSS scales may be regarded as a quick screening tool for dementia, especially the rNSS, which can be administered in less than 1 min.

## CONCLUSION

We confirmed an increase of NSSs in neurodegenerative dementias, compared to HCs. Patients with CBS or LBD exhibited the highest burden of NSSs compared to those with AD or FTD, possibly linked to greater involvement of sensorimotor areas and cerebellum. The development of a new, concise NSS Scale (rNSS) demonstrated promising diagnostic accuracy akin to the full Heidelberg NSS Scale, emphasizing extrapyramidal impairment. Our work adds to the wide literature on NSSs in diverse neuropsychiatric conditions, challenging the separation of “psychiatric” and “organic” diseases. NSS evaluation may represent a rapid screening tool for cognitive decline, particularly in differential diagnoses of neurodegenerative dementias.

## CLINICALTRIALS.GOV IDENTIFIER

NCT06354933.

## AUTHOR CONTRIBUTIONS

**Federico Emanuele Pozzi:** Conceptualization and design; acquisition and analysis of data; writing—drafting the manuscript; writing—review and editing. **Anna Falco:** Acquisition and analysis of data; writing—review and editing. **Gaia Gotti:** Acquisition and analysis of data; writing—review and editing. **Giuseppe Fiamingo:** Acquisition and analysis of data; writing—review and editing. **Giulia Remoli:** Acquisition and analysis of data; writing—review and editing. **Ildebrando Appollonio:** Writing—review and editing. **Carlo Ferrarese:** Writing—review and editing. **Lucio Tremolizzo:** Conceptualization and design; writing—review and editing.

## CONFLICT OF INTEREST STATEMENT

The authors declare no conflict of interest.

## ETHICS APPROVAL STATEMENT

The study protocol was approved by the ethical committee Comitato Etico Monza e Brianza, Italy; protocol DemeNSS, ID: 3785, and has therefore been performed in accordance with the ethical standards laid down in the 1964 Declaration of Helsinki and its later amendments.

## PATIENT CONSENT STATEMENT

N/A.

## CLINICAL TRIAL REGISTRATION

N/A.

## Supporting information

Supplementary Material ‐ results of the pilot work.

## Data Availability

The data of this manuscript are available upon request to the corresponding author.
