# Supplementary material for: Neurological soft signs in neurodegenerative dementias: Results of the DemeNSS study
Source: PCN Rep. 2025 Jun 25;4(2):e70143. doi: 10.1002/pcn5.70143 (PMC12188626; doi:10.1002/pcn5.70143)

**Supplementary Material – *Neurological Soft Signs: Italian version of Heidelberg Manual***

**Neurological Soft Signs in Neurodegenerative Dementias: Results of the DemeNSS Study**

Federico Emanuele Pozzi^1,2,3*^, Anna Falco^3^, Gaia Gotti^3^, Giuseppe Fiamingo^3^, Giulia Remoli^1,2,3^, Ildebrando Appollonio^1,2,3^, Carlo Ferrarese^1,2,3^, Lucio Tremolizzo^1,2,3^

^1^Neurology Department, Fondazione IRCCS San Gerardo, Monza, Italy

^2^Milan Center for Neuroscience (NeuroMI), University of Milano-Bicocca, Milan, Italy.

^3^School of Medicine and Surgery, University of Milano-Bicocca, Milan, Italy.

*F.E.Pozzi and A. Falco share first authorship*

*** Correspondence:**[f.pozzi26@campus.unimib.it](mailto:f.pozzi26@campus.unimib.it)

**Introduzione per l’esame**

L'esame deve essere eseguito in un ambiente tranquillo, senza interruzioni od ulteriori osservatori. I pazienti con storia di disturbi neurologici, abuso di droga o alcolismo vanno esclusi dall'esame. La procedura d’esame prevede che i primi test siano effettuati con il paziente in posizione eretta. Viene assegnato un punteggio in base alle capacità del paziente di svolgere un determinato esercizio:

1. Il paziente non ha difficoltà (o se le ha sono poco significative) nell’esecuzione dell'esercizio.
2. Problemi lievi, appena percepibili o intermittenti durante l’esercizio.
3. Difficoltà riconoscibile nell’esecuzione dell'esercizio.
4. Marcata difficoltà, problema continuamente presente, o prestazione del tutto deficitaria.

In una prestazione altrimenti normale (punteggio = 0), chiare differenze di lateralità sono quantificate come punteggio = 1.

*1. Andatura*

L'andatura viene giudicata dopo aver percorso una distanza sufficiente, con il paziente che cammina al suo passo normale - quindi preferibilmente prima dell'esame, quando il paziente si dirige verso la stanza. Prestare attenzione alla dinamica del movimento, alla lunghezza del passo e alla coordinazione, così come a movimenti delle braccia esagerati, ridotti, o asimmetrici.

1. Nessun problema rilevante.
2. Ridotto o esagerato pendolarismo delle braccia; dopo diversi passi l'impressione generale è di un’andatura poco fluida o a tratti alterata.
3. Una o due caratteristiche alterano in maniera netta l’andatura, come ad esempio piccoli passi, passi troppo lunghi, minima oscillazione delle braccia, perdita di ritmo.
4. Cammino a passi incerti, trascinamento poco fluido, o forti difficoltà di coordinazione impediscono il cammino.

*2. Camminare su una linea retta*

Istruzioni date al paziente: "Posizionando un piede direttamente davanti all'altro, provi a camminare in linea retta." Se richiesto, l'esaminatore mostra lui stesso l'esercizio. La distanza percorsa deve essere di almeno tre metri. L'esercizio deve essere eseguito prima con gli occhi aperti e ripetuto con gli occhi chiusi. Possibili problemi: disturbi dell'equilibrio, percorso serpeggiante, piede non posizionato esattamente davanti all’altro.

1. Nessuna difficoltà con gli occhi aperti. Con gli occhi chiusi solo poche difficoltà di equilibrio che vengono facilmente corrette.
2. L'esercizio è ben eseguito con gli occhi aperti. L’esaminando perde l'equilibrio con gli occhi chiusi e/o compie passi al di fuori della linea retta.
3. La persona perde l'equilibrio con gli occhi chiusi, ma con gli occhi aperti dimostra solo piccoli problemi.
4. Anche con gli occhi aperti notevoli problemi di equilibrio, deviazioni di percorso e passi troppo lunghi.

*3. Orientamento destra-sinistra*

Il paziente e l’esaminatore sono uno di fronte all'altro, ad una distanza pari alla lunghezza delle braccia. Istruzioni verbali date al paziente:

a) "Tocchi il suo braccio destro con il dito indice sinistro" b) "Tocchi il suo pollice destro con il dito indice sinistro"

c) "Tocchi la mia mano destra"

d) "Tocchi la mia mano sinistra - e ora la mia mano destra "

Laddove in (d) l'esaminatore tiene le braccia incrociate davanti al petto.

1. (a) e (b) completati senza esitazioni - in (c) e (d) soltanto una piccola esitazione.
2. Lieve esitazione in (a) o (b). Immediata auto-correzione di errori in (c) o (d).
3. Lunga incertezza in (a) o (b); spontanea auto-correzione in (a) o (b); un'azione scorretta in (c) o (d).
4. Errore già nella semplice indicazione in (a) o (b).

Nel caso in cui nei test successivi si osservi un problema di orientamento destra/sinistra, questo dovrebbe essere valutato basandosi sulla scala sopra indicata.

*4. Test delle braccia distese*

Il test è mostrato dall’esaminatore, che spiega: "Si metta con le gambe unite, le braccia in avanti, i gomiti estesi, palme verso l'alto, le dita divaricate, e chiuda gli occhi." Prestare attenzione allo slivellamento delle braccia, alla perdita di divaricazione delle dita, ai movimenti di opposizione dei mignoli, alla pronazione, e alle differenze di lato.

1. Entro un tempo ragionevole (1 min) non appare nessun problema.
2. Un lieve errore, ad esempio movimenti delle dita, o lieve pronazione, o leggera tendenza a lasciare cadere le braccia.
3. I difetti di (1) sono combinati e/o più pronunciati.
4. La posizione assunta cambia in maniera netta dopo pochi secondi. Diversi difetti combinati, ad esempio chiara caduta delle braccia, movimenti delle dita, pronazione.

*5. Prova indice-naso*

Subito dopo la prova numero 4: "Tenendo gli occhi chiusi, tocchi la punta del naso con il dito indice della mano destra, poi con il dito indice della mano sinistra." Se l’esaminando non è in grado di eseguire l'esercizio con gli occhi chiusi, dovrebbero essere eseguite due prove con occhi aperti.

1. L'esercizio viene eseguito tempestivamente e con sicurezza.
2. Lievi movimenti di correzione, o lieve imprecisione nell’indicazione, come ad esempio toccare il lato del naso con gli occhi chiusi.
3. Con gli occhi chiusi si verificano tremori di indecisione e/o inaccuratezza nell’indicazione. Con gli occhi aperti, tuttavia, l'esercizio è eseguito con facilità.
4. Anche con gli occhi aperti si verificano imprecisioni e tremori.

*6. Test di Ozeretzki*

I movimenti del test sono dimostrati e spiegati dall'esaminatore e poi eseguiti dal paziente assieme al medico per alcune volte.

Istruzioni: "Tenga le braccia protese in avanti, faccia un pugno con la mano destra e apra la mano sinistra; ora porti le braccia al suo petto e faccia due pugni con entrambe le mani; estenda le braccia di nuovo e apra bene la sua mano destra; continui a ripetere questa azione, aprendo le mani in modo alternato"

L'esercizio è svolto dapprima con gli occhi aperti e poi con gli occhi chiusi, dato che in questo modo le prestazioni migliorano nella maggior parte. Il paziente dovrebbe puntare alla maggior velocità possibile. Solitamente, l'esercizio deve essere mostrato più volte. Prestare attenzione alla velocità e al ritmo, esitazioni, ripartenze, e se le braccia sono completamente distese .

1. Dopo un po’ di pratica, l'esercizio viene eseguito con successo .
2. A velocità più elevate, solo brevi tratti di esercizio sono completati con successo, e questi sono interrotti da errori delle mani, perdita di ritmo , o il rallentamento della velocità.
3. Pur lentamente, solo alcune parti sono completate con successo. Difficoltà nel coordinare le braccia - il modello di base è, tuttavia, corretto .
4. Anche dopo una dimostrazione lenta, nessuna sezione è completata correttamente; bassa correlazione con le azioni mostrate.

*7. Diadococinesia*

L'esercizio viene spiegato e dimostrato: le mani sono sollevate ai lati della testa. Istruzioni: "Immagini che si stia sostituendo due lampadine, avvitandone una, e svitandone un'altra in senso contrario." Assicurare, correggendo e ri-dimostrando se necessario, che entrambe le mani possano pronare e supinare contemporaneamente. L'esercizio deve essere eseguito con sempre maggior velocità, prima con gli occhi aperti e poi con gli occhi chiusi. Prestare attenzione al ritmo, alle posizioni delle dita, alla sincronizzazione, alla velocità, e alle differenze di ampiezza tra i due lati.

1. L'azione è rapida e fluida. Al massimo si verifica lieve asimmetria (sviluppata dal lato dominante)
2. Difetti lievi, ad esempio perdita di ritmo, dita piegate - in particolare il quarto o quinto dito.
3. Diversi errori, ad esempio interruzioni, progressivo cambiamento nelle posizioni delle mani.
4. La performance si deteriora rapidamente dopo pochi secondi; difficoltà di coordinazione molto pronunciata, anche lentamente i movimenti sono aritmici.

*8. Pronazione-supinazione*

L'esercizio viene dimostrato e spiegato: in piedi, il paziente sbatte alternativamente il dorso e il palmo di una mano sul palmo dell'altra. Questo movimento che ricorda un applauso deve essere effettuato inizialmente con gli occhi aperti, e quindi con gli occhi chiusi. Il paziente deve puntare a svolgere l’esercizio alla massima velocità.

1. Prestazione veloce, sicura. Un leggero rallentamento sul lato non dominante non è significativo.
2. A velocità elevata, si verificano errori occasionali o pause, o le mani non sono completamente ruotate. Le prestazioni migliorano quando viene eseguito lentamente.
3. Difficoltà ed errori minori a bassa velocità.
4. Errori grossolani frequenti.

*9. Opposizione pollice-dita*

L'esercizio è dimostrato e spiegato al paziente, che ora viene fatto sedere: "Posi il dorso delle mani sulle cosce. Dapprima, con la mano destra, tocchi il pollice una volta con ogni dito, uno dopo l'altro, quindi inverta la sequenza delle dita." Inizialmente, se si verificano errori, spiegare e dimostrare l'esercizio di nuovo. Con gli occhi aperti, l'esercizio viene eseguito con la destra, e poi con la mano sinistra. L’esercizio deve essere ripetuto poi con gli occhi chiusi. Il paziente deve mirare alla prestazione più rapida possibile.

1. La performance è fluente e sicura.
2. Ritmo interrotto, lento; differenze di lato; singoli errori
3. Esitazioni e ripartenze; la sequenza delle dita non è corretta.
4. Anche lentamente, l'esercizio non può essere completato.

*10. Movimenti speculari*

Se durante l'esercizio 9 la mano passiva viene spostata, questi movimenti “a specchio” devono essere considerati come un'alterazione del sito attivo. Per esempio: mentre si esercita la mano destra, a sinistra appaiono i movimenti speculari, essi devono essere registrati come movimenti speculari destri.

1. Nessun movimento speculare percepibile.
2. Lieve contrazione attiva che appare solo ad intermittenza. (Movimenti trasferiti passivamente o tremori sono privi di significato)
3. Frequente, chiaro movimento di singole dita.
4. Movimenti simili a quelli descritti eseguiti dalla mano attiva.

*11. Discriminazione tra due punti*

La prova è eseguita utilizzando un calibro. La prova viene spiegata al paziente e mostrata sul suo braccio. Per evitare l’affaticamento della capacità discriminante dovuta alla ripetuta applicazione del calibro, si testano la punta del pollice e la punta delle dita. La pinza è posta, con delicatezza e per poco tempo, ad angolo retto rispetto alla lunghezza delle dita. Se le capacità discriminanti delle dita sono dissimili, viene presa quella del dito indice. In primo luogo, vengono utilizzati degli intervalli chiaramente discriminabili, quindi intervalli di 3, 4, 5, 6, ecc millimetri fino a che due punti ben distinti possono essere percepiti. L'affidabilità del test viene controllata tramite un intervallo di 0 mm.

1. Soglia minore o uguale a 3,5 mm.
2. Minore o uguale a 6mm.
3. Minore o uguale a 10mm.
4. Maggiore di 10 mm.

*12. Grafestesia*

Viene presentato al paziente un foglio di carta, su cui sono disegnate la lettera X, un cerchio, un quadrato, e il numero 3. Uno dei simboli è tracciato sulla sua mano, visto dal paziente. Egli dovrebbe cercare di riconoscere il simbolo. Se egli non è in grado di riconoscere il simbolo, nonostante l’aiuto visivo, il test non può essere valutato (può essere assegnato il punteggio "9 = non in grado"). Il paziente chiude gli occhi. Utilizzando una penna, la seguente sequenza di simboli viene tracciata (circa 3 centimetri di altezza) con delicatezza sul palmo del paziente: nel palmo della mano destra un quadrato; nel palmo della mano sinistra una X; a destra un cerchio; un quadrato a sinistra; a destra una X; a sinistra un 3; a destra un cerchio; a sinistra un 3.

1. Tutti i simboli sono facilmente riconoscibili.
2. Indecisione; errori immediatamente auto-corretti; un solo errore.
3. Due o tre errori.
4. Più di tre errori.

*13. Test faccia-mano*

Il paziente pone le palme delle mani sulle cosce. L'esaminatore si siede di fronte e spiega che lui toccherà il paziente in uno o due punti contemporaneamente e che il paziente dovrà dire da che lato e dove è stato toccato. Il paziente chiude gli occhi e, usando una penna in ciascuna mano, l'esaminatore lo tocca (leggermente e brevemente) in primo luogo sulla guancia destra, poi sulla mano sinistra, poi sulla guancia sinistra, e poi sulla mano destra. Tocca quindi il lato destro della fronte e la mano destra - poi il lato sinistro della fronte e la mano sinistra. Dopo 5 secondi di pausa entrambe le guance, e poi entrambe le mani, vengono toccate contemporaneamente con la stessa pressione. Di solito un paziente è meno in grado di individuare un doppio tocco, così, al primo errore nel doppio tocco, al paziente viene chiesto: "Solo lì? " Se si corregge da solo, non si tiene conto dell'errore.

1. 0 : Tutti i tocchi riconosciuti facilmente .
2. 1 : Un doppio tocco non viene riconosciuto .
3. 2 : Da 2 a 3 errori nel doppio tocco.
4. 3 : 4 o più errori, in particolare al singolo tocco.

*14. Stereoagnosia*

La prova è eseguita con tre monete: 1 centesimo, 5 centesimi, 50 centesimi. Al paziente viene detto che deve cercare di riconoscere tre monete poste singolarmente nella mano destra, e poi nella mano sinistra. Le monete non sono mostrate al paziente in anticipo. Sei prove sono condotte per ogni mano, nella sequenza:

a) destra: 1, 50, 5, 50, 1, 5

b) sinistra: 5, 1, 50, 5, 50, 1

Nel caso il paziente veda una delle monete, il test viene condotto con le monete da 5, 50, e 20 centesimi, o con le monete da 1, 2 e 20 centesimi. Il fattore decisivo per la valutazione non è il riconoscimento corretto del valore reale della moneta, ma la costanza nel riconoscimento della stessa moneta, anche quando questa sia stata denominata in modo scorretto la prima volta.

1. Tutte le monete sono coerentemente riconosciute. Massimo un errore per mano.
2. Scambio di monete di dimensioni simili (ad esempio 1 e 5 centesimi)
3. Scambio di monete di chiare dimensioni diverse (ad esempio 1 e 50 centesimi), ma in parte corretta identificazione.
4. Le monete non sono riconosciute. Il paziente indovina approssimativamente.

*15. Test di Luria (pugno-taglio-palmo)*

L’esercizio è dimostrato e spiegato: in primo luogo viene leggermente battuto il pugno prono sul tavolo, quindi si batte con il bordo della mano aperta, poi con il palmo, poi di nuovo con il pugno, ecc L'esercizio si esegue con la mano sinistra e poi con la destra, inizialmente con gli occhi aperti poi con gli occhi chiusi. Bisogna puntare a svolgerlo con la maggior velocità possibile. Alla prima comparsa di errori, l'esercizio deve essere eseguito assieme paziente di nuovo.

1. Veloce, prestazioni senza problemi.
2. Lenta; pugno a volte colpito sul lato; pause.
3. Sequenza a volte non corretta.
4. Nessuna sezione completata correttamente.

*16. Discorso e articolazione*

Le caratteristiche del linguaggio dei pazienti vanno osservate durante l'intero esame; vengono infine aggiunti due scioglilingua:

a) «Trentatré trentini entrarono a Trento tutti e trentatré trotterellando.»

b) «Sopra la panca la capra campa, sotto la panca la capra crepa.»

Ogni frase è pronunciata dall'esaminatore, poi ripetuta due volte da parte del paziente. Su richiesta, la frase viene ripetuta. Alla presenza di difficoltà, il test viene riavviato dopo una breve pausa.

1. Le frasi vengono ripetute rapidamente e fluidamente.
2. Singoli errori di pronuncia; errori in una sola sillaba rimangono anche alla seconda ripetizione.
3. Diversi errori nelle sillabe; cambia l’ordine delle parole.
4. Frase chiaramente alterata.

**Supplementary Material - *Results of the pilot work***

**Neurological Soft Signs in Neurodegenerative Dementias: Results of the DemeNSS Study**

Federico Emanuele Pozzi^1,2,3*^, Anna Falco^3^, Gaia Gotti^3^, Giuseppe Fiamingo^3^, Giulia Remoli^1,2,3^, Ildebrando Appollonio^1,2,3^, Carlo Ferrarese^1,2,3^, Lucio Tremolizzo^1,2,3^

^1^Neurology Department, Fondazione IRCCS San Gerardo, Monza, Italy

^2^Milan Center for Neuroscience (NeuroMI), University of Milano-Bicocca, Milan, Italy.

^3^School of Medicine and Surgery, University of Milano-Bicocca, Milan, Italy.

*F.E.Pozzi and A. Falco share first authorship*

*** Correspondence:**[f.pozzi26@campus.unimib.it](mailto:f.pozzi26@campus.unimib.it)

The sample was comprised by 29 healthy controls (with the same criteria listed below) and 85 patients with neurodegenerative dementias, of which 62 were affected by AD, 10 by FTD and 13 by either CBS or LBD. Patients were significantly older than controls (73.5 ± 7.5 vs 77.5 ± 6.0, p <0.01); as expected they exhibited significantly lower MMSE and FAB (17.9 ± 6.3 vs 29.4 ± 0.9 and 9.1 ± 4.7 vs 17.7 ± 0.9 respectively, p<0.01).

Patients exhibited higher rNSS-2 values than controls (12.4 ± 4.1 vs 4.2 ± 2.0, p<0.01, see **Table S1** and **Figure S1**).

Patients with FTD were significantly younger than other diagnostic groups (FTD: 66.6 ± 6.1, AD: 79.0 ± 4.3, CBS/LBD: 78.8 ± 4.3, p<0.01) and patients with AD had significantly lower education (FTD: 11.2 ± 5.3, AD: 6.9 ± 3.8, CBS/LBD: 10.2 ± 4.9, p<0.01). FTD patients had significantly higher MMSE (FTD: 22.5 ± 6.4, AD: 17.2 ± 5.9, CBS/LBD: 17.6 ± 6.7, p<0.05), while FAB and NPI were not significantly different among groups.

rNSS-2 was lower in patients with FTD, intermediate in patients with AD and higher in CBS/LBD (FTD: 8.2 ± 4.1, AD: 12.4 ± 3.9, CBS/LBD: 15.4 ± 2.7, p<0.01; see **Table S2** and **Figure S1**).

In linear models (**Table S3** and **Table S4**), being a patient, having lower MMSE and lower FAB were significant predictors of higher NSS on rNSS-2; moreover, among patients, being diagnosed with CBS or LBD and again exhibiting lower MMSE and FAB were significant predictors of higher rNSS-2 values. In controls, only having lower FAB was a significant predictor of higher rNSS-2 (**Table S5**); adding age and gender to linear models in controls resulted in loss of significance of the model.

A cut-off of 8.5 points on rNSS-2 distinguished patients from controls with a sensitivity of 100% and a specificity of 81.2% (AUC 95.3, 95% CI 91.8-98.8, **Figure S2**), while a cut-off of 11.5 points divided patients with CBS/LBD from patients with AD/FTD with a sensitivity of 60.4% and a specificity of 92.3% (AUC 82.6, 95% CI 73.0-92.1, **Figure S3**).

A significant positive correlation between rNSS-2 and age was seen in the whole sample and patients, but not in controls (**Figure S4**), while a significant correlation of rNSS-2 with education was only seen in the whole sample (**Figure S5**).

A ceiling effect of NSS around this value was observed with the rNSS-2. In particular, in 12 severe subjects (MMSE 8.7 ± 2.5, FAB 2.8 ± 1.7) we observed a mean rNSS-2 of 16.7 ± 1.7, with no significant correlation with MMSE (**Figure S6-S7**).

**Table S1**. Differences in baseline measures by group, patients vs controls.

| **Variable** | **Controls (n=29)** | **Patients (n=85)** | **p value** |
| --- | --- | --- | --- |
| Sex (F) | 65.5% | 54.1% | ns |
| Age | 73.5 ± 7.5 | 77.5 ± 6.0 | < 0.01 |
| Education | 8.9 ± 4.4 | 7.9 ± 4.4 | ns |
| MMSE | 29.4 ± 0.9 | 17.9 ± 6.3 | < 0.01 |
| FAB | 17.7 ± 0.9 | 9.1 ± 4.7 | < 0.01 |
| NPI | - | 17.0 ± 15.5 | - |
| rNSS-2 | 4.17 ± 2.0 | 12.4 ± 4.1 | < 0.01 |

**Table S2.** Differences in rNSS-2 by diagnostic group.

| **Variable** | **FTD (n =10)** | **AD (n=62)** | **CBS/LBD (n=13)** | **p** |
| --- | --- | --- | --- | --- |
| Sex (F) | 40% | 58.1% | 46.2% | ns |
| Age | 66.6 ± 6.1 | 79.0 ± 4.3 | 78.8 ± 4.3 | < 0.01 |
| education | 11.2 ± 5.3 | 6.9 ± 3.8 | 10.2 ± 4.9 | < 0.01 |
| MMSE | 22.5 ± 6.4 | 17.2 ± 5.9 | 17.6 ± 6.7 | < 0.05 |
| FAB | 12.0 ± 5.1 | 8.8 ± 4.8 | 8.1 ± 3.8 | ns |
| NPI | 24.5 ± 19.6 | 16.0 ± 15.8 | 15.6 ± 8.6 | ns |
| rNSS-2 | 8.2 ± 4.1 | 12.4 ± 3.9 | 15.4 ± 2.7 | < 0.01 |

**Table S3.** Linear model for rNSS-2 (whole sample, n =114, model p < 0.001).

|  | **rNSS2** | |
| --- | --- | --- |
| ***Predictors*** | ***Estimates*** | ***p*** |
| (Intercept) | 12.48 (5.33 – 19.64) | **0.001** |
| Sex [M] | -0.59 (-1.59 – 0.41) | 0.242 |
| Group [Patients] | 1.98 (0.43 – 3.52) | **0.013** |
| MMSE | -0.30 (-0.42 – -0.17) | **<0.001** |
| FAB | -0.31 (-0.48 – -0.14) | **<0.001** |
| Age | 0.07 (-0.01 – 0.15) | 0.078 |
| Education | 0.10 (-0.01 – 0.22) | 0.081 |
| R^2^ / R^2^ adjusted | 0.775 / 0.763 | |

**Table S4.** Linear model for rNSS-2 in patients (n=85, model p < 0.001).

|  | **rNSS2** | |
| --- | --- | --- |
| *Predictors* | *Estimates* | *p* |
| (Intercept) | 18.51 (9.09 – 27.92) | **<0.001** |
| Sex [M] | -0.77 (-1.90 – 0.37) | 0.182 |
| Diagnosis group [AD] | 2.39 (-0.00 – 4.78) | 0.050 |
| Diagnosis group [CBS or LBD] | 4.98 (2.39 – 7.57) | **<0.001** |
| MMSE | -0.30 (-0.43 – -0.17) | **<0.001** |
| FAB | -0.29 (-0.46 – -0.11) | **0.002** |
| Age | -0.02 (-0.15 – 0.11) | 0.773 |
| NPI | 0.01 (-0.03 – 0.05) | 0.663 |
| Education | 0.12 (-0.03 – 0.27) | 0.103 |
| R^2^ / R^2^ adjusted | 0.675 / 0.641 | |

**Table S5.** Linear model for rNSS-2 in controls (n=29, model p = 0.03).

|  | **rNSS2** | |
| --- | --- | --- |
| *Predictors* | *Estimates* | *p* |
| (Intercept) | 27.75 (4.74 – 50.76) | **0.020** |
| MMSE | -0.25 (-1.06 – 0.57) | 0.539 |
| FAB | -0.89 (-1.72 – -0.06) | **0.037** |
| education | -0.07 (-0.24 – 0.09) | 0.370 |
| R^2^ / R^2^ adjusted | 0.288 / 0.203 | |

**Figure S1.** Distribution of rNSS-2 across diagnostic groups.


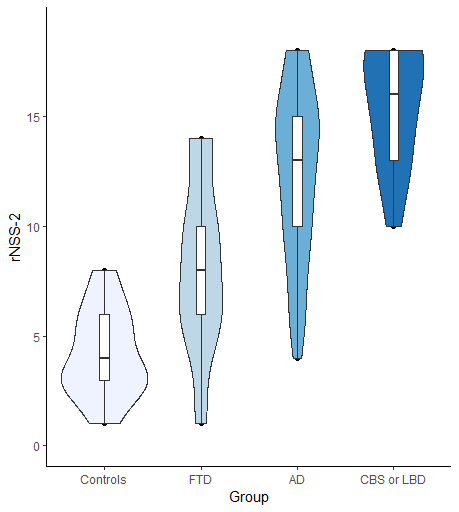


**Figure S2.** ROC curve for the diagnosis of neurodegenerative dementias vs controls, rNSS-2.


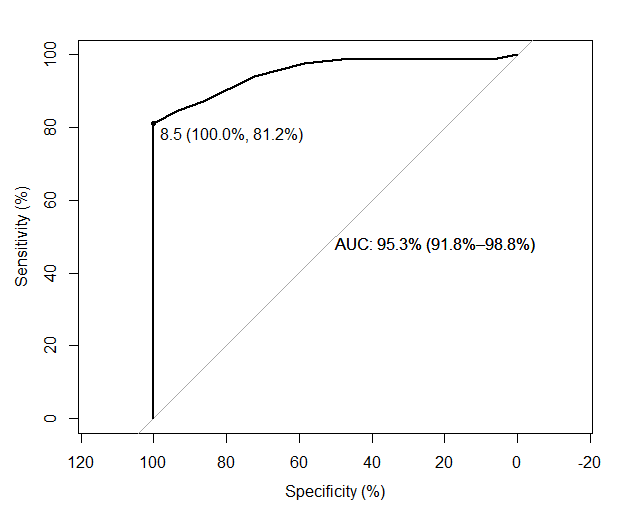


**Figure S3.** ROC curve for the diagnosis of CBS or LBD vs AD or FTD, rNSS-2.


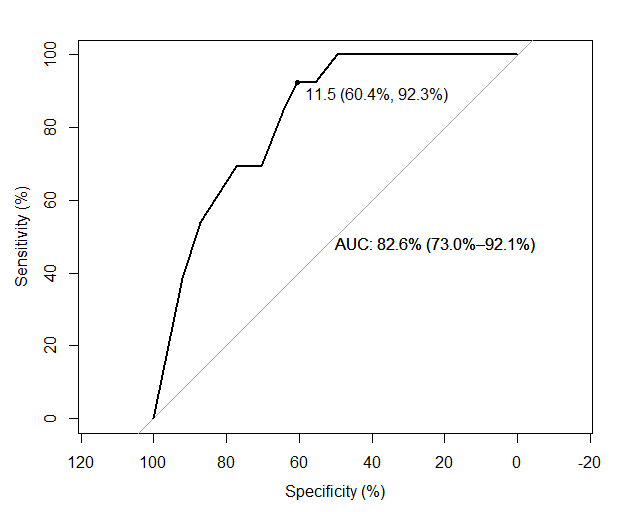


**Figure S4.** Correlation plot between rNSS-2 and age (whole sample, r=0.41, p<0.001). The correlation was significant only in patients (r=0.37, p<0.001), but not in controls (r=0.27, p=0.14).


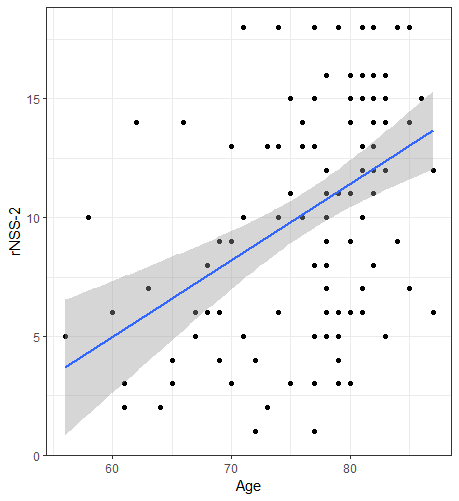


**Figure S5.** Correlation plot between rNSS-2 and education (whole sample, r=-0.18, p=0.044). The correlation was significant only in the whole sample (patients: r=-0.15, p=0.16; controls r=-0.30, p=0.11).


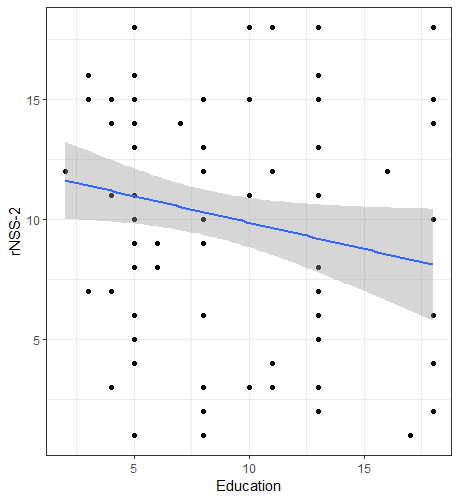


**Figure S6.** Correlation plot between rNSS-2 and MMSE in severely impaired patients.


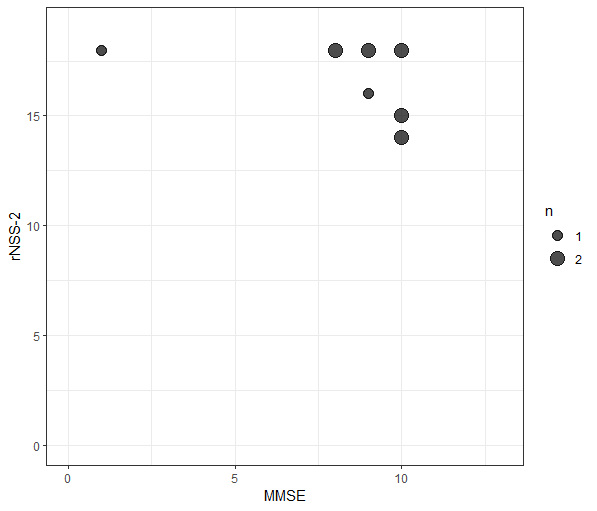


**Figure S7.** Correlation plot between rNSS-2 and FAB in severely impaired patients.


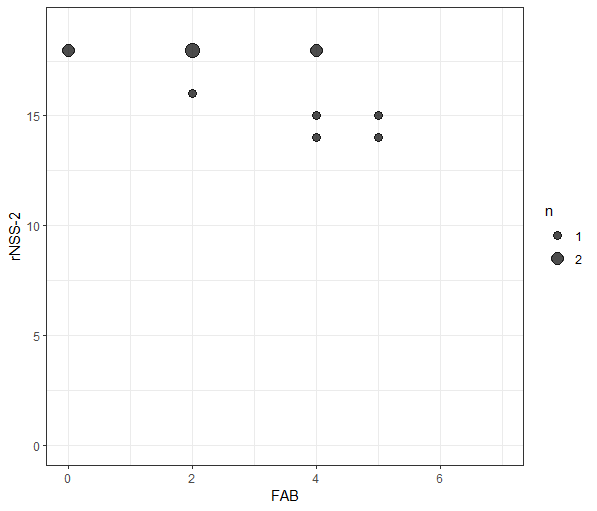

Supplement: Supplementary file 1 — Supplementary Material ‐ results of the pilot work. [file PCN5-4-e70143-s001.docx]
